# A stranger in a strange land: the utility and interpretation of heterologous expression

**DOI:** 10.3389/fpls.2015.00734

**Published:** 2015-09-15

**Authors:** Elena M. Kramer

**Affiliations:** Department of Organismic and Evolutionary Biology, Harvard University, Cambridge, MA, USA

**Keywords:** evo-devo, heterologous expression, functional evolution, biochemical evolution, developmental genetics

## Abstract

One of the major goals of the modern study of evodevo is to understand the evolution of gene function across a range of contexts, including sub/neofunctionalization, co-option of genetic modules, and the evolution of morphological novelty. To these ends, comparative studies of gene expression can be useful for constructing hypotheses, but cannot provide direct evidence of functional evolution. Unfortunately, determining endogenous gene function in non-model species is often not an option. Faced with this dilemma, a common approach is to use heterologous expression (HE) in genetically tractable model species as a proxy for functional analyses. Such experiments have important limitations, however, and require caution in the interpretation of their results. How do we dissociate biochemical function from its original genomic context? In the end, what does HE actually tell us? Here, I argue that HE only sheds light on specific types of biochemical conservation, but can be useful when experiments are carefully interpreted.

As developmental biologists, it is important to remember that when we speak of “gene function,” we are conflating, by necessity, a complex array of different factors. At a fundamental level, we can think of gene function as representing two complementary components: the first being biochemical function and the second being developmental role (Figure 1). The former is determined by the coding sequence of the gene itself and encompasses everything from secondary/tertiary protein structure, to enzymatic capacity, to co-factor and/or DNA binding site affinity. These aspects of gene function may change as the sequence of your favorite gene (YFG) itself evolves. As if this weren’t complicated enough, the actual developmental role played by YFG is the product of all of these primary components interacting with a wide array of *cis*- and *trans*-acting phenomena, including the expression patterns of YFG in relation to its co-factors, the epigenetic state of target loci, the position of binding sites within the genome, post-translation regulation of all interacting proteins, etc. Obviously, these secondary components evolve as well, to varying degrees in a coordinated fashion with YFG. So when we talk about the evolution of gene function, we are really considering the evolution of the whole genomic context of YFG—its protein sequence, *cis*- and *trans*-regulation, interacting partners, and target gene repertoire.

**FIGURE 1:**
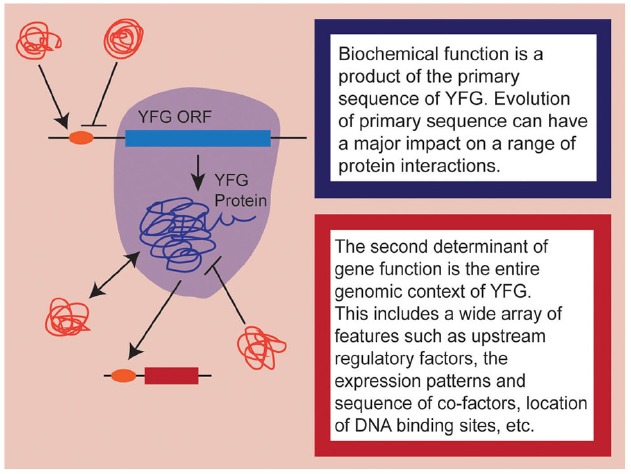
**A schematic representation of the dual nature of gene function.** Aspects influencing biochemical function are highlighted in shades of blue while aspects of the genomic context are highlighted in shades of red. Note that here, I am only considering heterologous expression of coding sequences, so upstream regulatory elements are considered to be part of the endogenous genomic context.

Heterologous expression (HE) takes the primary component of gene function—the sequence of the coding region itself—and plugs it into the second component—the genomic context—of a different species. We are essentially performing a site-directed mutagenesis experiment in which we ask whether the sequence differences between YFG and its endogenous homolog disrupt the functional roles normally played by the endogenous locus in its own genomic environment. Of course, HE can be conducted with varying degrees of rigor. The most rigorous approach is to drive expression with the endogenous promoter and ask whether YFG can rescue the phenotype of a null mutation in the endogenous locus. With surprising frequency, however, the heterologous locus is simply over-expressed in a wild type background (e.g., Lee et al., 2012; Perilleux et al., 2013; Lovisetto et al., 2015), such that the real question being asked is: Can this alien protein perturb development in the same manner as the endogenous protein when it is over-expressed? Such an approach creates new problems, including the nature of protein interactions, which are subject to reaction equilibria and therefore sensitive to the concentrations of the interacting factors.

Given this perspective, we should consider the variety of ways that HE is typically used in the evodevo field. These include to bolster evidence of genetic orthology (e.g., Serrano et al., 2009), to assess homology of a genetic module or an organ (e.g., Halder et al., 1995; Whipple et al., 2004), and to broadly assess conservation of gene “function” between taxa (e.g., Alvarez-Buylla et al., 2010; Kachroo et al., 2015). The first of these uses should be rejected since similarity of function is absolutely not a criterion for genetic homology in general or orthology in particular (Theissen, 2002; Gabaldon and Koonin, 2013). It is even true that positive HE results can be misleading when it comes to assessing orthology. Perhaps the best understood instance of this phenomenon is the *AGAMOUS* (*AG*) lineage of floral organ identity genes in flowering plants. The functions of *AG* homologs were first described in the core eudicot model systems *Arabidopsis* and *Antirrhinum* (snapdragon; Coen and Meyerowitz, 1991). In *Arabidopsis*, the *ag* mutant phenotype results in homeotic transformation of fertile organs into sterile organs and a loss of determinacy in the floral meristem. The *plena* (*ple*) mutant in *Antirrhinum* has the identical phenotype and *PLE* is clearly homologous to *AG*. However, *PLE* and *AG* are not orthologous but, rather, are derived from a whole genome duplication that occurred at the base of the core eudicots (Davies et al., 1999; Kramer et al., 2004). The orthologs of *PLE* in *Arabidopsis* are a pair of recent duplicates called *SHATTERPROOF1/2*, which participate in fruit and ovule development (Liljegren et al., 2000), while the ortholog of *AG* in *Antirrhinum* is called *FARINELLI* (*FAR*), a gene that primarily contributes to stamen development (Davies et al., 1999; Causier et al., 2005). These distinct functions appear to be due to independent patterns of subfunctionalization that occurred along the lineages leading to the rosid *Arabidopsis* on the one hand and the asterid *Antirrhinum* on the other. Furthermore, while the paralogs AG and PLE are biochemically equivalent in *Arabidopsis*, the orthologs AG and FAR are not (Causier et al., 2005; Airoldi et al., 2010). This is most likely due to changes in selection as *FAR* became specialized to function in stamen identity. Therefore, while it may commonly be true that orthologs are more likely than not to have both functional similarity and biochemical conservation, we cannot take it for granted.

If you will permit me a digression, I would also like to strongly discourage the common use of the term “functional ortholog.” It is important to remember that function is generally considered not to be a criterion for homology, even among genes (Theissen, 2002). I actually agree with Mindell and Meyer (2001) on this point, that there should be some leeway for discussing the inheritance of genetic function, but we should recognize that it is widely held that functions of any kind cannot be homologous. What information are we trying to convey when we say “functional ortholog?” We want to say that we have a pair of genes that are genetic orthologs and also appear to play similar functional roles. This is an important piece of information; certainly, we often want to know if function is conserved among orthologs. However, this terminology seems to suggest that “functional” orthologs have an additional quality of greater orthology because they show conserved function. This is simply untrue. Orthology is a feature of genetic relationship, of inheritance and patterns of gene duplication. It does not increase or decrease based on functional similarity. It is much more informative to say that you have performed a rigorous phylogenetic and/or syntenic analysis and have determined that the genes in question are orthologs and, further, appear to share conserved functions. We must recognize that this statement can really only be made if you have conducted endogenous functional studies in the taxa being compared. If you have only performed HE, then the best you can say is that there is some degree of biochemical conservation.

The use of HE to assess homology of a genetic module or an organ is more complex and relates to the need to distinguish between process homology and morphological homology, which has been well-covered by many previous authors (Bolker and Raff, 1996; Abouheif, 1997; Abouheif et al., 1997). These authors recognized quite early during the molecular renaissance of our field that shared expression of genetic homologs, and even shared developmental control by homologous genetic modules, should not be used as the basis for assessment of morphological homology. Hodin (2000) succinctly addressed the issue while discussing the limited value of HE with *Pax6* homologs: “A positive result tells you only that the biochemical properties of the protein have been conserved, not necessarily that its function within a certain morphological structure has also been conserved. The commonplace use of the same gene within an organism performing distinct functions in a multitude of tissue reveals why this experiment is generally uninformative with respect to evolutionary history (see also Abouheif et al., 1997).” Here, Hodin seeks to highlight the fact that conservation of biochemical interactions within a particular genetic module does not inform on the myriad of ways in which that module can be developmentally deployed. In this regard, I should note that HE can provide some relevant information if you are simply trying to assess homology of a genetic module, but I would argue that phylogeny-based homology assessment of the genes involved and tests of endogenous regulatory interactions are even more useful.

Process homology is especially relevant to cases of co-option of genetic modules to novel developmental functions. For instance, in butterflies *Distal-less* (*Dll*) orthologs have been recruited to promote the development of wing spots (Brunetti et al., 2001). The wing spot developmental program is very unlikely to be recapitulated by simply expressing the butterfly *Dll* in *Drosophila* because this developmental program is a product of what I defined as the second component of gene function, the endogenous genomic architecture of the butterfly. However, reciprocal HE of *Dll* orthologs between *Drosophila* and butterflies would be perfectly useful if your goal was to determine whether the evolution of the wing spot involved biochemical divergence in the butterfly *Dll* sequence. This type of co-option is just one extreme on a spectrum of evolutionary change that could also include morphological remodeling events such as the derivation of halteres from hindwings (Hersh et al., 2007), lodicules from petals (Whipple et al., 2007; Yoshida, 2012) or staminodia from stamens (Sharma and Kramer, 2013). Such evolutionary transitions may involve biochemical changes in upstream transcription factors but clearly also involve changes in target gene repertoires (e.g., Hersh et al., 2007). HE is much more likely to shed light on any biochemical changes rather than changes in target gene repertoires, which primarily depend on the positions of downstream binding sites dispersed throughout the genome.

The third common use of HE, to investigate conservation of “function,” is perfectly legitimate in many cases but less so in others. It is probably useful to start with a consideration of what can go wrong with HE. For instance, a lack of rescue or the failure to produce a phenotype may simply be due to the divergence between your species of interest and the reference model system. Even proteins that are likely to serve conserved functions can experience the process of developmental system drift (True and Haag, 2001) at the level of primary sequence. In other words, this is a site directed mutagenesis experiment in which the altered protein cannot function in the model system’s genomic context but may be perfectly functional in its original environment. On occasion, HE results in novel or dominant negative phenotypes (e.g., Lee et al., 2012; Katahata et al., 2014; Sun et al., 2014). These may be due to the disruptive effects of an alien protein being introduced to a system for which it is not adapted. If the heterologous protein can interact with some co-factors but not others, it may act as a dominant negative allele, especially when over-expressed. Perhaps most surprisingly though, even positive results can be misleading. Zarrinpar et al. (2003) tested the ability of SH3-domain protein homologs to rescue the function of one specific family member in yeast. They found that while endogenous paralogs were highly functionally specific and could not rescue, diverse metazoan homologs showed higher frequency of rescue. These results reflect the fact that members of the same genome, especially when co-expressed, will tend to co-evolve for a high degree of functional specificity. Homologs from divergent genomic contexts that have not experienced the same patterns of co-evolution may actually be quite promiscuous in a heterologous genome. Thus, we see that a range of results from HE can be uninformative or misleading, especially when you do not have functional data from the original organism.

So am I suggesting that HE is never useful for examining the evolution of gene function? Certainly not. In cases where biochemical divergence is specifically being assessed, this approach can be the best experiment to use, albeit with some caveats. Let’s consider a classic HE experiment, Ronshaugen et al. (2002), in which they tested the ability of *Artemia* Ubx to suppress limb development in *Drosophila* (Figure 2). Interestingly, the authors found that while the full length *Artemia* Ubx had little limb-suppressing capacity, a relatively minor C-terminal deletion allowed the *Artemia* protein to repress limbs in *Drosophila*. In light of this finding, the authors proposed a model in which the Ubx protein of a crustacean/insect ancestor experienced mutation in the C-terminus of the protein that uncovered a limb-repression function. This is certainly a plausible scenario that fits the presented data, but we should also recognize a weakness in that the experiment was only performed in the *Drosophila* genomic context where Ubx has a limb repressing function. If you could put the mutated *Artemia* Ubx back into *Artemia*, would it have the capacity to repress limbs or is that primarily a product of the *Drosophila* genome? As it turns out, further studies in *Artemia* have revealed a more complex situation that suggests that there may be multiple reasons why Ubx does not repress limbs in *Artemia* (Hsia et al., 2010). These findings underscore the fact that accurate interpretation of HE data really hinges on having as much information as possible in both taxa, including functional results whenever possible.

**FIGURE 2:**
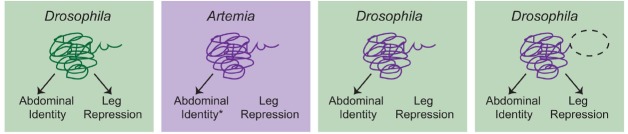
**A schematic overview of the Ronshaugen et al. (2002) experiments.** *Drosophila* Ubx promotes abdominal identity and acts to repress limb development in the abdomen. *Artemia* Ubx is assumed (*) to promote abdominal identity but not repress leg development. Consistent with this, when full-length *Artemia* Ubx was expressed in *Drosophila*, it was capable of promoting abdominal identity but could not repress leg development. Deletion of a C-terminal region of the *Artemia* Ubx (dashed oval) conferred leg repression capacity. This led to the conclusion that evolution of the C-terminal domain was a critical aspect of the evolution of leg repression function in the Ubx lineage in arthropods. However, there are several considerations that should be kept in mind. First, additional studies suggest that there may be multiple reasons why *Artemia* Ubx does not repress leg development (Hsia et al., 2010). Second, the ideal test would be to determine whether the truncated *Artemia* Ubx could repress legs if placed back into the endogenous *Artemia* context. Without this experiment, it remains possible that the observed function is simply a product of the *Drosophila* genomic context, in which Ubx normally represses leg development. Given everything we know now, the most conservative interpretation is that clearly *Artemia* Ubx is not biochemically equivalent to *Drosophila* Ubx. These biochemical differences may have been critical for the evolution of limb repressing functions, but studies in *Artemia* itself, as well as other arthropods would be necessary to confirm this hypothesis.

One especially elegant demonstration of how powerful HE can be when paired with functional studies in both the donor and recipient is work done on the control of flowering time in sugar beet (*Beta vulgaris* ssp. *vulgaris,* Pin et al., 2010). In flowering plants, homologs of the PEBP lineage defined by the *Arabidopsis* gene *FLOWERING LOCUS T* (*FT*) are broadly involved with promoting the transition from vegetative to reproductive development (reviewed Ballerini and Kramer, 2011). The FT protein has been identified as the classic Florigen factor that moves from leaves, where it is produced, to the apical meristem in order to change meristem identity. Consistent with this role, most *FT* homologs are only expressed at significant levels after the initiation of reproductive development. In cultivated sugar beet, however, a very recent gene duplication has given rise to two copies: *BvFT1*, which is primarily expressed during vegetative development, and *BvFT2*, which is expressed as expected during the reproductive stage (Pin et al., 2010). Using RNAi and overexpression in beet, Pin et al. (2010) clearly established that the *BvFT1* paralog had acquired a dominant negative effect that represses flowering until vernalization (cold treatment) represses *BvFT1* and allows expression of the floral promoting paralog *BvFT2*. This dramatic difference in function between the two paralogs can be recapitulated in *Arabidopsis*, where BvFT2 activates flowering while BvFT1 represses it. This demonstrates that there is a biochemical change in BvFT1 relative to the otherwise highly conserved function of FT proteins. The use of chimeric proteins and site-directed mutagenesis in the more tractable *Arabidopsis* system allowed the authors to identify the specific amino acid changes that are responsible for the neofunctionalization, and further demonstrate that these changes are associated with BvFT1 alleles that were selected during domestication. This kind of study relies heavily on HE but uses it in exactly the right way—by targeting an otherwise highly conserved genetic module, and in combination with detailed expression and functional studies in the original system, which allows the heterologous results to be accurately interpreted.

Another powerful application of HE is to use homologs from a series of diverging taxa to probe the conservation of specific biochemical properties, such as recognition of DNA binding sites. This is essentially a matter of letting evolution do the site-directed mutagenesis for you: as you move out to more deeply diverging taxa, there are more non-synonymous mutations, allowing you to ask whether the endogenous biochemical function is still retained. The land plant-specific transcription factor *LEAFY* (*LFY*) is ideal for this type of study because unlike most plant gene lineages, it has very few retained paralogs. Maizel et al. (2005), tested the ability of *LFY* homologs from across the land plants to rescue the *lfy* mutation in *Arabidopsis*, and then further complemented the phenotypic analysis with microarray studies of gene expression. They found that there was a gradual decreasing degree of phenotypic rescue as they moved out to more distantly related taxa. When paired with tests of protein/DNA interaction, their results suggest that “the declining ability to replace *Arabidopsis* LFY … is caused by a progressive failure to interact with the canonical LFY binding sites,” which, of course, are defined based on work done in *Arabidopsis*. The microarray analysis of the various transgenic lines demonstrated that in the weakest cases of rescue, one of the last target interactions to be lost was with the floral meristem identity gene *APETALA1* (*AP1*). The authors quite correctly noted that this finding does not tell us anything about what the heterologous LFY homologs activate in their endogenous settings—*AP1* homologs are not even present outside angiosperms. Rather, this reflects the extraordinarily high affinity of the LFY binding site present in the *AP1* promoter, such that even deeply divergent homologs with many non-synonymous changes are still capable of recognizing it. This kind of study highlights evolutionary processes affecting both aspects of developmental gene function since it detects biochemical changes that have altered DNA affinity while also underscoring the fact that repertoires of target genes will simultaneously be evolving.

In summary, my argument is that HE can be very useful in specific cases where we want to investigate changes in the primary component of gene function, which is to say biochemical function. This includes enzymatic capacity as well as affinity for a range of interactions such as protein-DNA and protein–protein. It yields the best results when paired with functional studies in both the donor and recipient taxa so that potentially spurious phenotypes can be ruled out. I think it is also true that HE works best when you can target a genetic module that is otherwise very highly conserved, so that you can lessen the impact of drift and divergence in other components of the pathway (although this is hard to ever rule out completely!). HE does not inform upon homology in general or orthology in particular, nor does it give us much information on what developmental roles the gene may play in its original genomic context, so use it with care.

## Conflict of Interest Statement

The author declares that the research was conducted in the absence of any commercial or financial relationships that could be construed as a potential conflict of interest.

## References

[B1] Abouheif E. (1997). Developmental genetics and homology: a hierarchical approach. Tren. Ecol. Evol. 12, 405–408. 10.1016/S0169-5347(97)01125-7 21238133

[B2] Abouheif E. Akam M. Dickinson W. J. Holland P. W. H. Meyer A. Patel N. H. (1997). Homology and developmental genes. Tren. Gen. 13, 432–433. 10.1016/S0168-9525(97)01271-7 9385839

[B3] Airoldi C. A. Bergonzi S. Davies B. (2010). Single amino acid change alters the ability to specify male or female organ identity. Proc. Natl. Acad. Sci. U.S.A. 107, 18898–18902. 10.1073/pnas.1009050107 20956314 PMC2973880

[B4] Alvarez-Buylla E. R. Ambrose B. A. Flores-Sandoval E. Vergara-Silva F. Englund M. Garay-Arroyo A. (2010). B-function expression in the flower center underlies the homeotic phenotype of *Lacandonia schismatica* (Triuridaceae). Plant Cell 22, 3543–3559. 10.1105/tpc.109.069153 21119062 PMC3015125

[B5] Ballerini E. S. Kramer E. M. (2011). In the light of evolution: a reevaluation of conservation in the CO-FT regulon and its role in photoperiodic regulation of flowering time. Front. Plant Sci. 2:81. 10.3389/fpls.2011.00081 22639612 PMC3355682

[B6] Bolker J. A. Raff R. A. (1996). Developmental genetics and traditional homology. Bioessays 18, 489–494. 10.1002/bies.950180611 8787536

[B7] Brunetti C. R. Selegue J. E. Monteiro A. French V. Brakefield P. M. Carroll S. B. (2001). The generation and diversification of butterfly eyespot color patterns. Curr. Biol. 11, 1578–1585. 10.1016/S0960-9822(01)00502-4 11676917

[B8] Causier B. Castillo R. Zhou J. L. Ingram R. Xue Y. B. Schwarz-Sommer Z. (2005). Evolution in action: following function in duplicated floral homeotic genes. Curr. Biol. 15, 1508–1512. 10.1016/j.cub.2005.07.063 16111944

[B9] Coen E. S. Meyerowitz E. M. (1991). The war of the whorls: genetic interactions controlling flower development. Nature 353, 31–37. 10.1038/353031a0 1715520

[B10] Davies B. Motte P. Keck E. Saedler H. Sommer H. Schwarz-Sommer Z. (1999). PLENA and FARINELLI: redundancy and regulatory interactions between two *Antirrhinum* MADS-box factors controlling flower development. EMBO J. 18, 4023–4034. 10.1093/emboj/18.14.4023 10406807 PMC1171478

[B11] Gabaldon T. Koonin E. V. (2013). Functional and evolutionary implications of gene orthology. Nat. Rev. Genet. 14, 360–366. 10.1038/nrg3456 23552219 PMC5877793

[B12] Halder G. Callaerts P. Gehring W. J. (1995). Induction of ectopic eyes by targeted expression of the eyeless gene in *Drosophila*. Science 267, 1788–1792. 7892602 10.1126/science.7892602

[B13] Hersh B. M. Nelson C. E. Stoll S. J. Norton J. E. Albert T. J. Carroll S. B. (2007). The UBX-regulated network in the haltere imaginal disc of *D. melanogaster*. Dev. Biol. 302, 717–727. 10.1016/j.ydbio.2006.11.011 17174297 PMC1892158

[B14] Hodin J. (2000). Plasticity and constraints in development and evolution. J. Exp. Zool. (Mol. Dev. Evol.) 288, 1–20. 10.1002/(SICI)1097-010X(20000415)288:1<1::AID-JEZ1>3.0.CO;2-7 10931501

[B15] Hsia C. C. Pare A. C. Hannon M. Ronshaugen M. Mcginnis W. (2010). Silencing of an abdominal Hox gene during early development is correlated with limb development in a crustacean trunk. Evol. Dev. 12, 131–143. 10.1111/j.1525-142X.2010.00399.x 20433454 PMC2893884

[B16] Kachroo A. H. Laurent J. M. Yellman C. M. Meyer A. G. Wilke C. O. Marcotte E. M. (2015). Systematic humanization of yeast genes reveals conserved functions and genetic modularity. Science 348, 921–925. 10.1126/science.aaa0769 25999509 PMC4718922

[B17] Katahata S. I. Futamura N. Igasaki T. Shinohara K. (2014). Functional analysis of SOC1-like and AGL6-like MADS-box genes of the gymnosperm *Cryptomeria japonica*. Tree Genet. Genomes 10, 317–327. 10.1007/s11295-013-0686-9

[B18] Kramer E. M. Jaramillo M. A. Di Stilio V. S. (2004). Patterns of gene duplication and functional evolution during the diversification of the AGAMOUS subfamily of MADS-box genes in angiosperms. Genetics 166, 1011–1023. 10.1534/genetics.166.2.1011 15020484 PMC1470751

[B19] Lee J. H. Park S. H. Ahn J. H. (2012). Functional conservation and diversification between rice OsMADS22/OsMADS55 and *Arabidopsis* SVP proteins. Plant Sci. 185, 97–104. 10.1016/j.plantsci.2011.09.003 22325870

[B20] Liljegren S. J. Ditta G. S. Eshed Y. Savidge B. Bowman J. L. Yanofsky M. F. (2000). SHATTERPROOF MADS-box genes control seed dispersal in *Arabidopsis*. Nature 404, 766–770. 10.1038/35008089 10783890

[B21] Lovisetto A. Baldan B. Pavanello A. Casadoro G. (2015). Characterization of an AGAMOUS gene expressed throughout development of the fleshy fruit-like structure produced by *Ginkgo biloba* around its seeds. BMC Evol. Biol. 15:139. 10.1186/s12862-015-0418-x 26173604 PMC4502469

[B22] Maizel A. Busch M. A. Tanahashi T. Perkovic J. Kato M. Hasebe M. (2005). The floral regulator LEAFY evolves by substitutions in the DNA binding domain. Science 308, 260–263. 10.1126/science.1108229 15821093

[B23] Mindell D. P. Meyer A. (2001). Homology evolving. Trends Ecol. Evol. 16, 434–440. 10.1016/S0169-5347(01)02206-6

[B24] Perilleux C. Pieltain A. Jacquemin G. Bouche F. Detry N. D’aloia M. (2013). A root chicory MADS box sequence and the *Arabidopsis* flowering repressor FLC share common features that suggest conserved function in vernalization and de-vernalization responses. Plant J. 75, 390–402. 10.1111/tpj.12208 23581257

[B25] Pin P. A. Benlloch R. Bonnet D. Wremerth-Weich E. Kraft T. Gielen J. J. L. (2010). An antagonistic pair of FT homologs mediates the control of flowering time in sugar beet. Science 330, 1397–1400. 10.1126/science.1197004 21127254

[B26] Ronshaugen M. Mcginnis N. Mcginnis W. (2002). Hox protein mutation and macroevolution of the insect body plan. Nature 415, 914–917. 10.1038/nature716 11859370

[B27] Serrano G. Herrera-Palau R. Romero J. M. Serrano A. Coupland G. Valverde F. (2009). *Chlamydomonas* CONSTANS and the evolution of plant photoperiodic signaling. Curr. Biol. 19, 359–368. 10.1016/j.cub.2009.01.044 19230666

[B28] Sharma B. Kramer E. M. (2013). Sub- and neofunctionalization of APETALA3 paralogs have contributed to the evolution of novel floral organ identity in *Aquilegia* (columbine, Ranunculaceae). New Phytol. 197, 949–957. 10.1111/nph.12078 23278258

[B29] Sun W. Huang W. J. Li Z. N. Song C. Liu D. Liu Y. L. (2014). Functional and evolutionary analysis of the AP1/SEP/AGL6 superclade of MADS-box genes in the basal eudicot *Epimedium sagittatum*. Ann. Bot. 113, 653–668. 10.1093/aob/mct301 24532606 PMC3936592

[B30] Theissen G. (2002). Secret life of genes. Nature 415, 741. 10.1038/415741a 11845189

[B31] True J. R. Haag E. S. (2001). Developmental system drift and flexibility in evolutionary trajectories. Evol. Dev. 3, 109–119. 10.1046/j.1525-142x.2001.003002109.x 11341673

[B32] Whipple C. J. Ciceri P. Padilla C. M. Ambrose B. A. Bandong S. L. Schmidt R. J. (2004). Conservation of B-class floral homeotic gene function between maize and *Arabidopsis*. Development 131, 6083–6091. 10.1242/dev.01523 15537689

[B33] Whipple C. J. Zanis M. J. Kellogg E. A. Schmidt R. J. (2007). Conservation of B class gene expression in the second whorl of a basal grass and outgroups links the origin of lodicules and petals. Proc. Natl. Acad. Sci. U.S.A. 104, 1081–1086. 10.1073/pnas.0606434104 17210918 PMC1783367

[B34] Yoshida H. (2012). Is the lodicule a petal: molecular evidence? Plant Sci. 184, 121–128. 10.1016/j.plantsci.2011.12.016 22284716

[B35] Zarrinpar A. Park S. H. Lim W. A. (2003). Optimization of specificity in a cellular protein interaction network by negative selection. Nature 426, 676–680. 10.1038/nature02178 14668868

